# Closed-Loop Efficient Searching of Optimal Electrical Stimulation Parameters for Preferential Excitation of Retinal Ganglion Cells

**DOI:** 10.3389/fnins.2018.00168

**Published:** 2018-03-19

**Authors:** Tianruo Guo, Chih Yu Yang, David Tsai, Madhuvanthi Muralidharan, Gregg J. Suaning, John W. Morley, Socrates Dokos, Nigel H. Lovell

**Affiliations:** ^1^Graduate School of Biomedical Engineering, UNSW Sydney, Sydney, NSW, Australia; ^2^Biological Sciences, Columbia University, New York, NY, United States; ^3^Electrical Engineering, Columbia University, New York, NY, United States; ^4^School of Aerospace, Mechanical and Mechatronic Engineering, University of Sydney, Sydney, NSW, Australia; ^5^School of Medicine, Western Sydney University, Penrith, NSW, Australia

**Keywords:** ON and OFF RGCs, frequency and amplitude modulation, preferential activation, retinal prostheses, closed-loop optimization

## Abstract

The ability for visual prostheses to preferentially activate functionally-distinct retinal ganglion cells (RGCs) is important for improving visual perception. This study investigates the use of high frequency stimulation (HFS) to elicit RGC activation, using a closed-loop algorithm to search for optimal stimulation parameters for preferential ON and OFF RGC activation, resembling natural physiological neural encoding in response to visual stimuli. We evaluated the performance of a wide range of electrical stimulation amplitudes and frequencies on RGC responses *in vitro* using murine retinal preparations. It was possible to preferentially excite either ON or OFF RGCs by adjusting amplitudes and frequencies in HFS. ON RGCs can be preferentially activated at relatively higher stimulation amplitudes (>150 μA) and frequencies (2–6.25 kHz) while OFF RGCs are activated by lower stimulation amplitudes (40–90 μA) across all tested frequencies (1–6.25 kHz). These stimuli also showed great promise in eliciting RGC responses that parallel natural RGC encoding: ON RGCs exhibited an increase in spiking activity during electrical stimulation while OFF RGCs exhibited decreased spiking activity, given the same stimulation amplitude. In conjunction with the *in vitro* studies, *in silico* simulations indicated that optimal HFS parameters could be rapidly identified in practice, whilst sampling spiking activity of relevant neuronal subtypes. This closed-loop approach represents a step forward in modulating stimulation parameters to achieve appropriate neural encoding in retinal prostheses, advancing control over RGC subtypes activated by electrical stimulation.

## Introduction

Retinal neuroprostheses or bionic eyes, aim to restore functional visual percepts to those suffering from retinal degenerative diseases (Rizzo and Wyatt, 1997; Palanker et al., 2005; Weiland et al., 2005). With such devices, it is desirable to elicit visual percepts by activating retinal neuron populations in a controlled spatiotemporal pattern.

Human vision is primarily mediated by two major retinal ganglion cell (RGC) classes—the ON and OFF cells, which respond to an increase and decrease in light intensity, respectively. If these RGCs can be selectively or preferentially activated in a desired temporospatial sequence, more physiologically-realistic patterns of neural activity could be elicited by a neural prosthesis. However, closely distributed ON and OFF RGCs are often simultaneously activated during electrical stimulation, due to the spatial mismatch between the clinical stimulation electrode and small receptive field of human midget RGCs, resulting in ambiguous encoding of visual information. This may be a major contributor of unexpected percepts reported in clinical trials (Humayun et al., 2003; Rizzo et al., 2003; Yanai et al., 2007; Zrenner et al., 2011; Sinclair et al., 2016). Using a combination of *in vivo* (Barriga-Rivera et al., 2017), *in vitro* (Jensen et al., 2005; Tsai et al., 2009, 2011; Weitz et al., 2015), and *in silico* studies (Dokos et al., 2005; Horsager et al., 2009; Tsai et al., 2012; Barriga-Rivera et al., 2017), researchers have been working toward a better understanding of how these clinically reported percepts might arise, including how RGC activation can be achieved with high spatiotemporal accuracy (Tsai et al., 2009; Freeman et al., 2011; Guenther et al., 2012). While the underlying cause(s) are topics of ongoing research, we reasoned that by obeying more physiological coding scheme of the visual system, through preferential stimulation of neuronal types, visual information can be conveyed and interpreted more accurately by the brain, thereby improving the quality of the evoked percepts.

High-frequency electrical stimulation (HFS), which we define as being from 0.1 to 12 kHz, has been used extensively in auditory-nerve stimulation to improve the performace of cochlear prosthetics (Zierhofer et al., 1995; Huang and Shepherd, 1999; Litvak et al., 2001, 2003; Cai et al., 2011, 2013; Twyford et al., 2014). For example, Litvak and colleagues found that HFS up to 5 kHz can generate more stochastic firing in auditory nerve fibers (Litvak et al., 2001, 2003). Furthermore, recent retinal stimulation studies have suggested the possibility of using HFS to target functionally-distinct RGC types. For example, an *in vitro* study conducted by Cai et al. (2011) indicated that functionally-identified RGCs responded differentially to electrical stimuli when stimulated at frequencies ranging from 0.1 to 0.7 kHz. More recent *in vitro* studies by Twyford et al. (2014) and Cai et al. (2013) suggested that it may be possible to preferentially activate different RGC subtypes using 2 kHz HFS applied in close proximity to the RGCs' axon initial segment (AIS). These *in vitro* results were further studied using *in silico* approaches (Guo et al., 2014; Kameneva et al., 2016).

Previous reports indicated that appropriate HFS-based neuromodulation may elicit preferential excitation of different RGCs in a manner similar to RGC responses to light stimuli in a healthy retina. To test the hypothesis that HFS neuromodulation is able to preferentially excite ON and OFF RGC pathways, we evaluated the performance of a range of electrical stimulation amplitudes (10–240 μA) and frequencies (1–6.25 kHz) on RGC responses using murine retinal preparations. HFS amplitude and frequency parameters were optimized to elicit RGC responses resembling those evoked by visual stimuli.

We also developed a closed-loop optimization algorithm using simulated neural responses as real-time feedback, to effectively search for stimulation parameters that maximize the stimulation current range for preferential activation of ON and OFF RGC populations, providing a method to rapidly probe the responses of ON and OFF RGCs over a broad range of stimulus parameters.

## Materials and methods

### Animal and retinal whole-mount preparation

WT C57BL/6 mice (aged 4–8 weeks, purchased from Australian BioResource) were anesthetized with 4% vaporized isoflurane delivered into an induction chamber and euthanized by cervical dislocation. Both eyes were enucleated and placed in Ames' medium (Sigma-Aldrich, St. Louis, MO) with 1% penicillin/streptomycin (Invitrogen) and equilibrated with carbogen (5% CO_2_/95% O_2_). Each eye was hemisected and the intact retina isolated from the retinal pigment epithelium. The vitreous humor was removed to reduce the curvature of the retina and aid in flattening its surface. Four equally spaced radial cuts from the brim of the retina to the center were made, to dissect it into four pieces. Each piece was then placed photoreceptor-side down on a modified Millicell Biopore filter membrane insert (PICM01250, Millipore, Billerica, MA, USA), and secured onto the filter membrane in accordance with Toychiev et al. (2013). The retinal piece along with the filter membrane insert was then transferred to an imaging chamber (RC-40HP, Warner Instruments, Hamden, CT, USA), where the glass-bottom of the chamber was smeared with Vaseline such that the filter membrane insert could be held steadily. Axonal bundles were observed under IR light to locate the optic nerve, which allowed us to orient the retina with the optic nerve located either at the top or bottom of the chamber. All procedures were reviewed and approved by the UNSW Animal Care and Ethics Committee and were carried out in compliance with the Australian Code of Practice for the Care and Use of Animals for Scientific Purposes.

The retinal piece was imaged using a 40x objective lens (LUMPlanFL N, Olympus, Tokyo, Japan) under a fixed-stage upright microscope (SliceScope, Scientifica, Uckfield, United Kingdom). We used an infra-red light source with peak wavelength of 780 nm and a full-width-at-half-maximum (FWHM) value of 25 nm. The light was transmitted through a DODT gradient contrast system to illuminate the retinal piece. We used a high sensitivity CMOS camera (DCC3240N, Thorlabs, Newton, NJ, USA) to continuously capture the images, which were displayed on an external monitor with ThorCam software (Thorlabs, Newton, NJ, USA). Patched RGCs were visualized using Alexa Fluor 488 dye with LED wavelength excitation at 470 nm (M470L3, Thorlabs, Newton, NJ, USA). Resulting epi-fluorescent images were captured using a scientific camera (1500M-GE, Thorlabs, Newton, NJ, USA).

### Whole-cell patch clamp recording with high-frequency extracellular electrical stimulation

For light stimulation, we illuminated the retinal piece with a white LED using reflecting mirrors (Thorlabs, Newton, NJ, USA) to guide the light through the condenser. A spot of light was centered over the soma of the patched RGC and its responses to the light stimulus were used to determine the RGC functional subtype as either ON or OFF.

Patch pipettes were pulled from borosilicate glass (Warner Instrument, Hamden, CT) with outer/inner diameters of 1.50/0.86 mm using a micropipette puller (Sutter Instrument, Novato, CA). The patch pipettes were filled with internal solution containing (in mM) 106 KMeSO_4_, 0.0078 CaCl_2_, 1 MgCl, 10 HEPES, 0.7 EGTA, 10 KCl, 10 Phosphocreatine-Na, 4 ATP-Na_2_, 0.5 GTP-Na_3_, and adjusted to pH 7.2 with KOH. All chemicals were purchased from Sigma-Aldrich. To visualize the patched RGCs and their axons and dendrites, we added 70 μM Alexa Fluor 488 hydrazide fluorescent dye (Thermo Fisher Scientific, Waltham, MA) into the internal solution. The patch pipette resistances ranged between 3 and 6 MΩ. All recordings were performed using a MultiClamp 700B amplifier (Molecular Devices, Sunnyvale, CA, USA). The data were low-pass filtered at 10 kHz and digitized at 50 kHz with a Digidata 1440A, pCLAMP 10 software (Molecular Devices, Sunnyvale, CA, USA). All data were analyzed in Matlab R2017b (Mathworks).

A cocktail of synaptic blockers was used to suppress synaptic inputs, mimicking late stage retinal degeneration, consisting of (in mM) 0.01 NBQX (2,3-Dioxo-6-nitro-1,2,3,4-tetrahydrobenzo[f]quinoxaline-7-sulfonamide) to block AMPA/kainate receptors, 0.05 D-AP5 [(2R)-amino-5-phosphonovaleric acid] to block NMDA receptors, 0.02 L-AP4 [L-(+)-2-Amino-4-phosphonobutyric acid] to block mGluR6, 0.1 picrotoxin (pic) to block GABA_a/c_ receptors and 0.01 strychnine (stry) to block glycinergic receptors. We confirmed synaptic blockade by the absence of RGC light responses. All pharmacological agents were purchased from either Sigma-Aldrich or Tocris Bioscience.

We delivered HFS using a STG 4002 stimulator (MultiChannel Systems hardware and software, Reutlingen, Germany), with a stimulation duration of 300 ms. Stimulation frequencies of 1.0, 1.67, 2.0, 2.5, 3.33, 4.17, 5.0, and 6.25 kHz were chosen in order to create approximate linear frequency steps. The sequence of frequencies delivered during each experiment was 1.0, 6.25, 1.67, 5.0, 2.5, 4.17, and 3.33 kHz in order to avoid possible effects of a monotonically changing stimulation frequency, if any. Both cathodic and anodic phases were 40 μs in duration, without any inter-phase interval (see **Figure 2A**). The extracellular stimulus ranged from 10 to 240 μA, in 10 μA steps. The inter-stimulation duration was set to be 2.7 s in order to allow all cells to fully recover from previous electrical stimulation. Each pulse amplitude was delivered three times. The mean spike-stimulus curve was calculated for each cell, and the overall mean was calculated again across the ON (*N* = 11) and OFF (*N* = 12) population, respectively. Standard error of mean (SEM) is calculated to estimate the precision of estimated mean of population-based RGC spike rates.

For each RGC, we defined a local 3D x, y, z coordinate system such that the upper surface of the RGC layer was aligned in the x-y plane and the RGC axon was aligned with the y-axis. The platinum-iridium stimulating electrode of diameter 25 μm was placed 40 μm from the soma at 2D coordinates of (0, −40) μm viewed from above, where (0, 0) μm are the local 2D coordinates of the soma. The stimulating electrode was initially placed on the upper surface of the ganglion cell layer (epi-retinal placement). To ensure consistent electrode positioning, we lowered the stimulating electrode until it touched the inner limiting membrane (ILM), and then raised it 20 μm vertically (z-axis). All stimulating electrode locations were controlled and recorded from the Sutter controller display panel (Sutter instrument, Novato, CA, USA). A Ag/AgCl reference electrode was placed in the bath ~2 cm away from the stimulation electrode (monopolar configuration). The stimulating electrode was moved along the x-y plane under whole-mount view.

### A closed-loop algorithm searching for optimal stimulation parameters

A simple empirical model was used to qualitatively describe experimental ON and OFF RGC spikes as a function of stimulation amplitude and frequency,

(1) $$\begin{array}{r} \sigma_{ON}\left(A,\;F\right)=\frac{\alpha_{ON}}{e^{\beta_{ON}\left(A-\gamma_{ON}\right)}+e^{-\delta_{ON}\left(A-\gamma_{ON}\right)}}\\ \sigma_{OFF}\left(A,\;F\right)=\frac{\alpha_{OFF}}{e^{\beta_{OFF}\left(A-\gamma_{OFF}\right)}+e^{-\delta_{OFF}\left(A-\gamma_{OFF}\right)}}+\varepsilon \end{array}$$

where σ_*ON*_ and σ_*OFF*_ represent the population-based ON and OFF total spike counts, which are amplitude- and frequency-dependent. α, β, γ, and δ are frequency-dependent functions, each with an intercept term and a multiplicative term (see Table 1). ε is a scalar parameter representing spontaneous spikes observed in the OFF RGC population. In total, 8 ON model parameters and 9 OFF model parameters were estimated to match the *in vitro* data observed in ON (*N* = 11) and OFF (*N* = 12) RGC populations from 12 mice. Model formulations and estimated parameter values are given in Table 1. In order to simulate physiological variations among different RGC populations, 17 empirical model parameters were randomly perturbed from their default values using values drawn from a uniform probability distribution, centered at the default value of each parameter, with a maximum parameter deviation of ±30%. The simulation was performed 15 times to generate 15 different sets of ON and OFF population with population-specific parameters.

**Table 1:** Estimated formulations and parameters for empirical model.

**Empirical ON and OFF cell model formulations and parameters**	
**ON**	**OFF**
$\sigma_{ON}\left(A,\;F\right)=\frac{\alpha_{ON}}{e^{\beta_{ON}\left(A-\gamma_{ON}\right)}+e^{-\delta_{ON}\left(A-\gamma_{ON}\right)}}$	$\sigma_{OFF}\left(A,\;F\right)=\frac{\alpha_{OFF}}{e^{\beta_{OFF}\left(A-\gamma_{OFF}\right)}+e^{-\delta_{OFF}\left(A-\gamma_{OFF}\right)}}+1$
α_*ON*_ = **29** − **11** × (*F* − 1)/5.25	α_*OFF*_ = **57** − **3** × (*F* − 1)/5.25
β_*ON*_ = **0.005** + **0.008** × (*F* − 1)/5.25	β_*OFF*_ = **0.012** + **0.042** × (*F* − 1)/5.25
γ_*ON*_ = **135** − **35** × (*F* − 1)/5.25	γ_*OFF*_ = **135** − **45** × (*F* − 1)/5.25
δ_*ON*_ = **0.068** + **0.002** × (*F* − 1)/5.25	δ_*OFF*_ = **0.03** + **0.012** × (*F* − 1)/5.25

*σ_ON_(A, F) and σ_OFF_(A, F) are the stimulus amplitude- and frequency-dependent averaged total spike numbers of ON and OFF cells. α, β, γ, and δ are frequency-dependent, each with an intercept term and a multiplicative term. α, β, γ, and δ are estimated to match the observed in vitro data. 17 estimated parameters (8 for ON model and 9 for OFF model) are shown in bold format. Units of β and δ functions are 1/μA, while units of the γ function are in μA. A is in μA. F is in kHz*.

A closed-loop searching algorithm using modeled ON and OFF RGC spikes as real-time feedback, was developed to automatically explore the optimal stimulus amplitude and frequency in different instances of virtual RGC populations. Preferential activation was achieved by simultaneously minimizing the electrically-evoked spike rate of ON cells whilst maximizing the spike rate of OFF cells, and *vice versa*. Formally, this involves minimizing the following objective functions:

(2) $$\begin{array}{r} \varphi_{ON}\;\left(A,\;F\right)=-\sigma_{ON}\left(A,\;F\right)/\left(\sigma_{OFF}\left(A,\;F\right)+1\right)\\ \varphi_{OFF}\;\left(A,\;F\right)=-\sigma_{OFF}\left(A,\;F\right)/\left(\sigma_{ON}\left(A,\;F\right)+1\right) \end{array}$$

where *A* ∈ (10, 240) μ*A*, and *F* ∈ (1, 6.25) kHz. All parameter searches began at (10 μA, 0.5 kHz).

**Figure 1:**
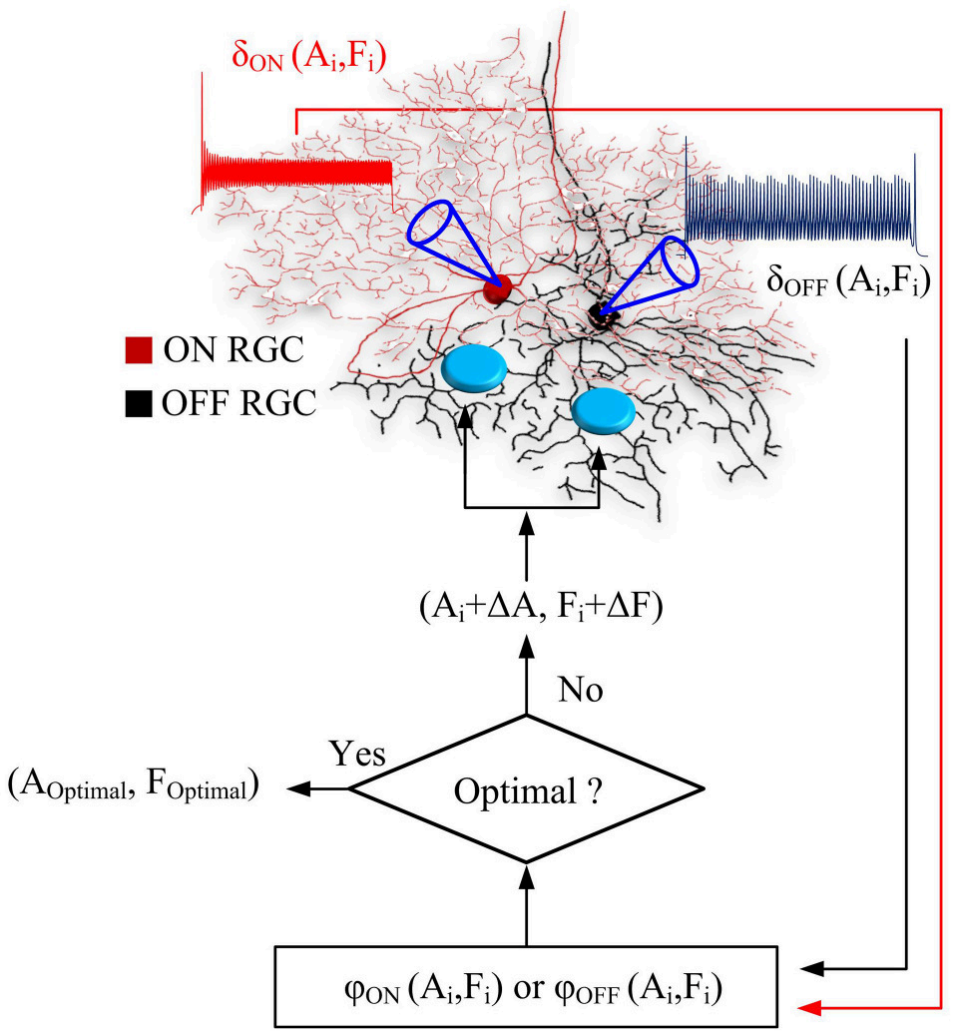
Flow chart of the real-time, closed-loop algorithm for optimal stimulus parameters. At each iteration, ON and OFF RGC spikes, σ_*ON*_(*A*_*i*_, *F*_*i*_) and σ_*OFF*_(*A*_*i*_, *F*_*i*_) elicited at newly-searched HFS amplitude and frequency *(Ai, Fi)* were computed and used to update the outputs of objective functions. If the test step *(Ai, Fi)* did not decrease the objective functions φ_*ON*_ and φ_*OFF*_, the algorithm rejected this test step, and pick a new test step *(Ai-1*+Δ*Ak, Fi-1*+Δ*Fk)*. Otherwise, the algorithm accepts this test step as part of the ongoing trajectory. The searching was terminated when the minimal values of φ_*ON*_(*A, F*) and φ_*OFF*_(*A, F*) were found in the given parameter space.

A flow chart in Figure 1 illustrated the process of optimal parameter searching. At each iteration, overall ON and OFF RGC spikes (σ_*ON*_ and σ_*OFF*_) elicited at newly-searched HFS amplitude and frequency were recorded to update objective functions. If the test step (*A*_*i*_*, F*_*i*_) did not decrease the objective functions φ_*ON*_ and φ_*OFF*_, the algorithm rejected this test step, and pick a new test step (*A*_*i*−1_ + Δ*A*_*k*_*, F*_*i*−1_ + Δ*F*_*k*_). Otherwise, the algorithm accepts this test step as part of the ongoing trajectory. The searching was terminated when the minimal values of φ_*ON*_(*A, F*) and φ_*OFF*_(*A, F*) were found in the given parameter space. An interior point algorithm (Byrd et al., 1999; Waltz et al., 2006) was used for searching the minimum of objective functions. An in-built function from MATLAB optimization toolbox namely FMINCON was used. All simulations were performed and analyzed in Matlab R2017b (Mathworks).

## Results

### Preferential RGC activation can be achieved by modulating stimulation amplitude and frequency *in vitro*

**Figure 2:**
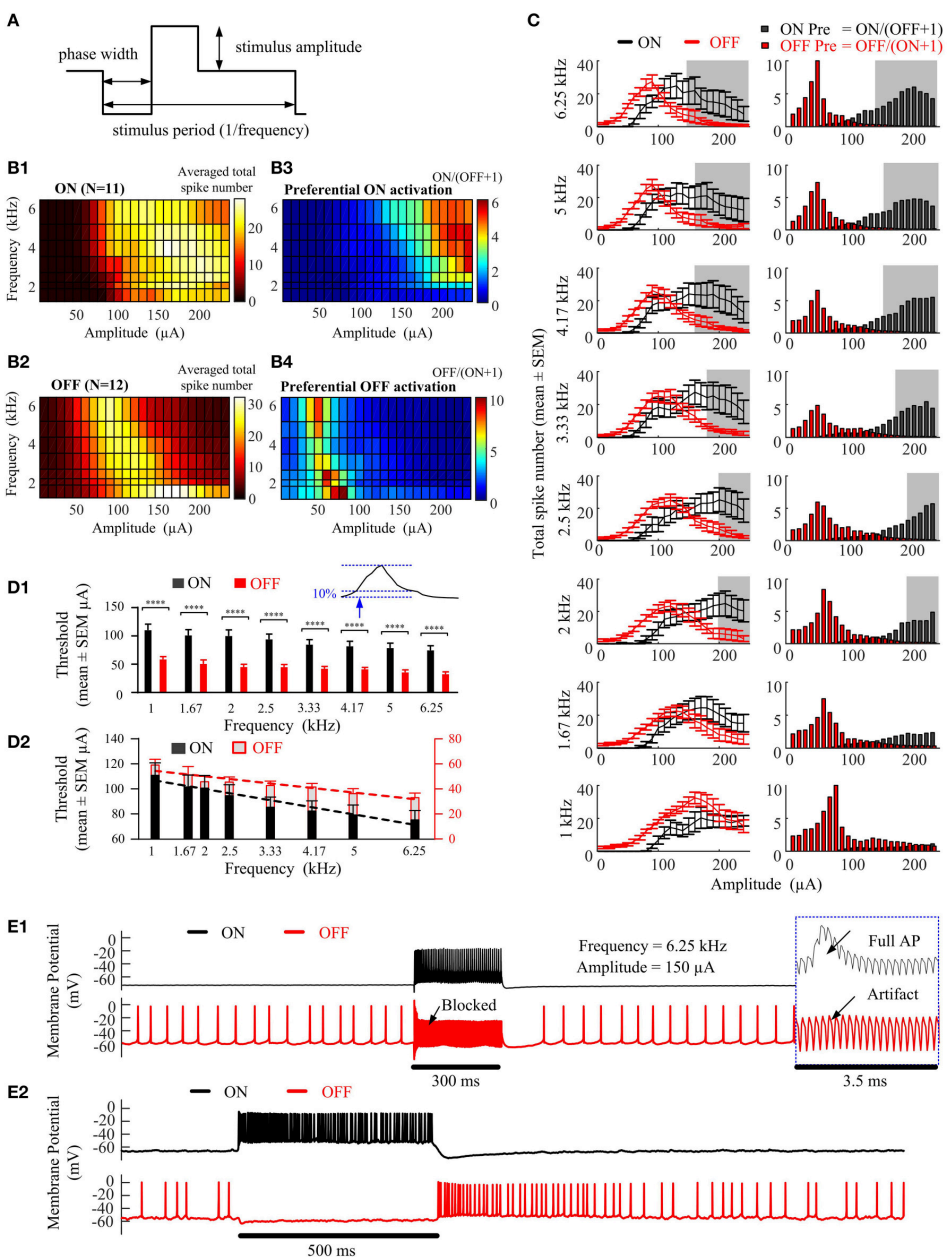
Preferential RGC activation as a function of stimulation amplitude and frequency. **(A)** High-frequency electrical stimulation waveform. Cathodic-first, charge-balanced, biphasic stimuli with pulse width of 40 μs per phase were used. The stimulus period was determined by the pulse repetition frequency. All pulse trains were 300 ms in duration. **(B1,B2)** Activation maps showing the averaged total spike number elicited in ON (*N* = 11) and OFF (*N* = 12) RGCs in response to a range of stimulation amplitudes (10~240-μA) and frequencies (1~6.25-kHz). All RGCs were identified using a stationary spot of light. **(B3,B4)** Preferential RGC excitability map is defined as the ratio of the averaged total spike numbers of ON RGCs over that of the OFF RGCs. **(C)** Left column, juxtaposition of the averaged ON and OFF spike count against stimulating amplitude, at frequencies 6.25, 5.0, 4.17, 3.33, 2.5, 2.0, 1.67, and 1.0 kHz. The error bars indicate standard error of the mean. Right column, Stimulus-dependent preferential ON and OFF activation at each stimulation frequency, indicated by the ratio of averaged total spike numbers of ON over that of the OFF cells. The shaded region shows the optimal stimulation settings for which the overall mean spike number of the ON cell population is three times that of the OFF cell population. i.e., σ_*ON*_(*A*_*i*_, *F*_*i*_) ≥ 3 σ_*OFF*_(*A*_*i*_, *F*_*i*_). **(D1)** Stimulation thresholds (defined by the stimulation amplitude able to elicit 10% of the maximum spike number of each non-monotonic spike-stimulus profile) for ON and OFF RGC populations. The ON cell population had a generally higher threshold across all stimulation frequencies than the OFF cell population (repeated measure 2-way ANOVA followed by multiple comparisons with Bonferroni correction, ^****^p < 0.0001). **(D2)** Linear regression lines fitted to stimulation thresholds against stimulation frequencies for ON and OFF populations. The negative values of the slopes of linear regression lines (−0.0067 ± 0.00076 for ON RGC population, and −0.0043 ± 0.00062 for OFF RGC population) indicated a trend of decreasing threshold with increasing frequency (*p* < 0.05). Different scales were used for ON (black) and OFF (red) RGC populations. **(E1)** Examples of RGC responses from the ON and OFF RGC populations in **(B)**. With 6.25 kHz stimuli of 150 μA, the ON RGC was strongly activated, while simultaneously blocking the OFF RGC's spontaneous spikes, yielding only stimulus artifacts. Insert: expanded view of the traces. **(E2)** Examples of ON and OFF RGC responses following light stimulation. The HFS-induced neuromodulation in **(E1)** elicits preferential excitation of ON and OFF RGCs in a manner similar to RGC responses to light stimuli in the healthy retina **(E2)**.

In Figure 2, we explored the ability of epiretinal HFS to preferentially activate ON (*N* = 11) and OFF (*N* = 12) RGC populations obtained in 12 mice. Figures 2B1,B2 show the number of spikes elicited in ON and OFF RGCs in response to a range of stimulation frequencies (1–6.25 kHz) and amplitudes (10–240 μA). The colors denote the number of evoked spikes for a given stimulation frequency and pulse amplitude (Figure 2A), averaged across all recorded ON (Figure 2B1) or OFF cells (Figure 2B2). All elicited spikes were recorded at the soma after application of the synaptic blockers. The total average spike number of ON cells reached a plateau as the stimulus current increased above a certain threshold at frequencies of 1, 2, and 2.5 kHz. However, with frequencies higher than 3.33 kHz, the averaged total spike number increased initially with stimulus amplitude, followed by a decline with further amplitude increase, creating a non-monotonic surface in the frequency-amplitude topological space (Figure 2B1). The OFF cells also exhibited a non-monotonic surface at all tested stimulation frequencies.

The preferential activation maps in Figures 2B3,B4 highlight the stimulation frequencies and amplitudes for preferentially activating ON and OFF RGCs. Each grid point was determined from the ratio of total spike numbers for one cell type verses the other. That is, ON/(OFF+1) for ON preferential activation and OFF/(ON+1) for OFF preferential activation. We found that preferential activation of ON RGCs was maximized at relatively higher stimulation amplitudes (>150 μA) and frequencies (>2 kHz). In contrast, HFS pulse trains across all frequencies were able to induce robust preferential activation of OFF RGCs with stimulation amplitudes between 40 and 90 μA. Moreover, the threshold at which preferential activation began for both cell types gradually reduced as the stimulus frequency was increased from 1 to 6.25 kHz. The stimulation current range for preferentially activating ON RGCs increased when the stimulating frequency increased from 2 to 6.25 kHz, while the stimulation current range for preferentially activating OFF RGC was mostly stable across all frequencies.

The left column of Figure 2C shows the averaged ON and OFF stimulus-dependent response curves with standard error bar at each frequency for comparison. With increasing stimulation frequency, both ON and OFF RGCs exhibited an increased slope of the rising phase in spikes/μA (the phase in which spike counts increase with increasing stimulation current) and concomitantly, an earlier onset of the falling phase (in which the averaged total spike numbers saturate or decline).

The shaded region in Figure 2C shows the optimal stimulation settings for which the ON cell population can be strongly excited while minimizing spikes from the OFF population. Specifically, the optimal stimulation parameters were defined as the parameter space for which the overall ON cell spike number is three times that of the OFF cell population, i.e., σ_*ON*_(*A*_*i*_, *F*_*i*_) ≥ 3 σ_*OFF*_(*A*_*i*_, *F*_*i*_). Additional results are provided in the Supplementary Figure 1 to show the preferential activation map of individual ON and OFF RGC pairwise (11x12).

Figure 2D demonstrates statistical analysis of differential stimulus-dependent response curves recorded from ON and OFF populations. Repeated measure 2-way analysis of variance (ANOVA) followed by multiple comparison with Bonferroni correction were used to test significant differences in stimulation thresholds. In Figure 2D1, the stimulation threshold was defined as the stimulation amplitude capable of eliciting 10% of the maximum spike number of the non-monotonic spike-stimulus profile measure at each frequency. The ON cell population had a generally higher threshold across all stimulation frequencies than the OFF cell population (^****^p < 0.0001). Moreover, the results indicated a trend of decreasing threshold with increasing frequency. Figure 2D2 shows linear regression lines fitted to stimulation thresholds against stimulation frequencies for ON and OFF populations. The negative values of the slopes of linear regression lines (Y = −0.006747X + 113.4 for ON RGC population, and Y = −0.004316X + 58.71 for OFF RGC population) indicated a decreasing stimulation threshold to HFS pulse trains by increasing stimulation frequency (non-zero slope significance *p* < 0.05).

To further confirm our findings, Figure 2E shows an example of optimal HFS-induced responses recorded by whole-cell patch clamp for a pair of mouse ON and OFF cells. In the presence of synaptic blockers, the ON RGC was silent, while the OFF RGC (8/12) showed only low-frequency spontaneous spikes (6.20 ± 0.13 Hz) (Margolis and Detwiler, 2007). Next, when the HFS was delivered at 6.25 kHz and 150 μA (black underlined segment, 300 ms), the ON RGC was strongly activated, while the OFF RGC was silent. Importantly, the OFF RGC spontaneous spikes were also inhibited (see also Figure 2E1 insert). The spontaneous OFF responses then recovered after offset of the stimulus. Figure 2E2 shows an example of ON and OFF RGC membrane potentials in response to 500 ms of light stimulation. The HFS-induced neuromodulation (Figure 2E1) elicited preferential excitation in a manner similar to the differential activation of ON and OFF RGCs that occurs physiologically following a light stimulus (Figure 2E2), and reported ON and OFF RGC response to light (Wang et al., 2001; Zhang et al., 2016; Tsai et al., 2017). All of these results illustrated the potential for artificial retinal stimulation to mimic natural RGC encoding.

### Optimizing stimulation parameters to maximize preferential activation using ON and OFF RGC responses as real-time feedback

To generate the preferential activation map of Figure 2B, 192 trials (8 frequencies: 6.25, 5.0, 4.16, 3.33, 2.5, 2.0, 1.67, 1.0 kHz, and 24 levels of amplitude: from 10 to 240 μA) of biphasic pulse trains with different stimulation parameter combinations had to be delivered to an individual RGC. The trial number will grow further as the stimulus parameter search space increases, requiring considerable demand on experimental resources and limiting result throughput. Therefore, we developed an automated algorithm to robustly and effectively find the optimal stimulation parameters without delivering all possible pulse trains. In addition, we investigated whether physiological variations between RGCs could significantly affect the utility of the algorithm.

**Figure 3:**
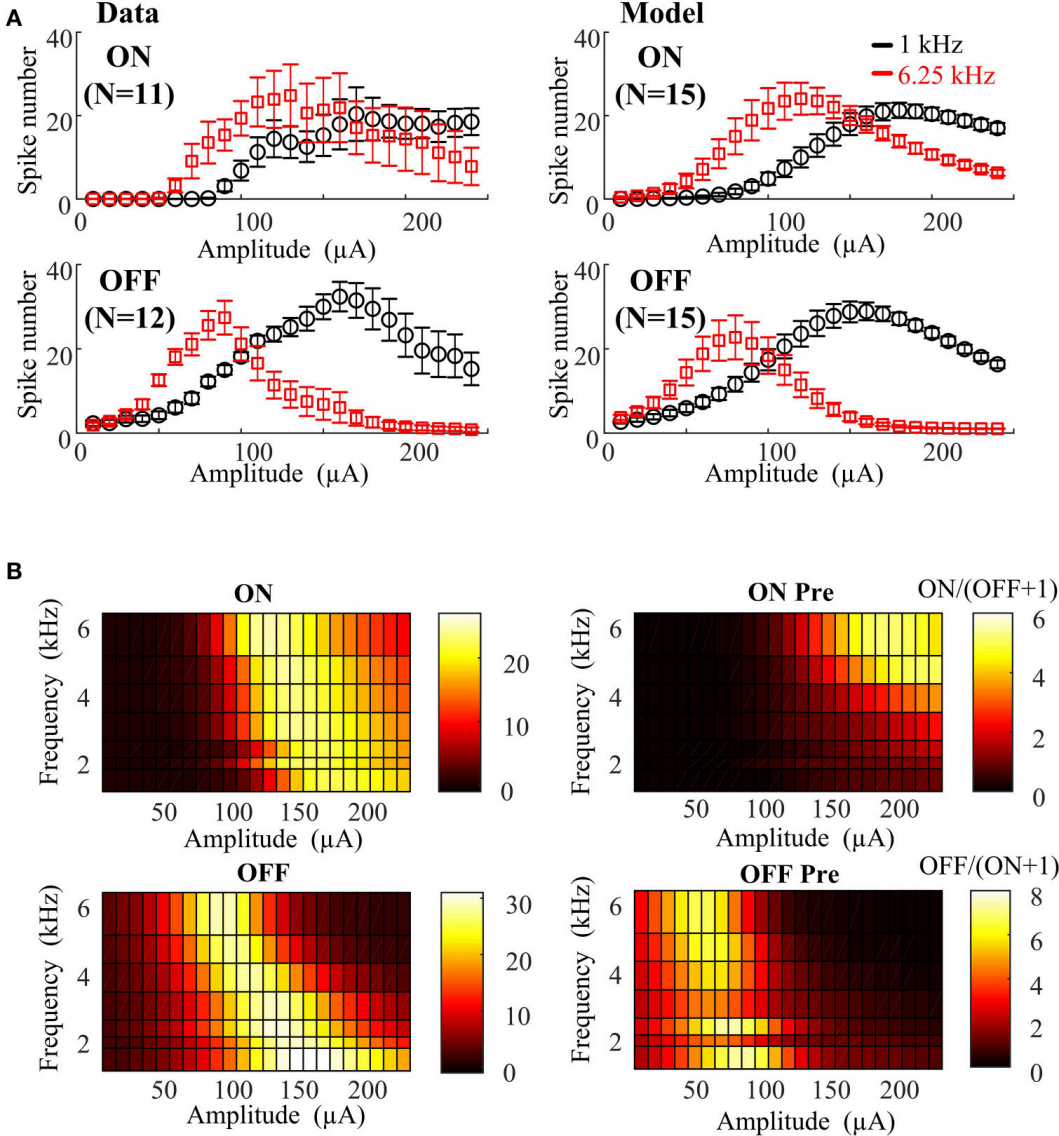
Empirical model closely replicating RGC activation observed experimentally under different stimulus frequencies and amplitudes. **(A)** Left panel: *In vitro* 1- and 6.25-kHz spiking responses against stimulus amplitude recorded in mouse ON (*N* = 11) and OFF (*N* = 12) RGCs with standard error bars. Right panel: 1- and 6.25-kHz spiking responses against stimulus amplitude simulated by the 15 virtual RGC populations. Fifteen sets of parameters were generated from a uniform random distribution centered around the default values. Maximum parameter deviations were set at ±30% of default values. **(B)** Stimulated RGC activation maps plotted based on mean values of the 15 model parameter sets.

As a first step to address these issues, we developed an empirical computational model to capture the effects of HFS on ON and OFF responses. The *in vitro* results can be quantitatively described using an empirical function of stimulation amplitude and frequency utilizing 17 free parameters (see Table 1). In order to assess the effect of physiological variations on the preferential RGC activation map, 15 different instances of ON and OFF RGC virtual populations were simulated with randomly perturbed model parameters. For each simulation, ON and OFF empirical model parameters were randomly perturbed from their default values using a uniform probability distribution centered around the default value of each parameter. Maximum parameter deviations were set at ±30% of the default value. The simulated ON and OFF RGC population responses (mean ± SEM) are shown in Figure 3, where it is evident that for ±30% maximum deviation, ON and OFF RGC behaviors were comparable to those observed *in vitro*. In Figure 3B, the model based on the 15 sets of RGC populations was able to closely replicate the actual RGC activation map observed in Figure 2A.

In the second step, we developed a parameter searching algorithm able to rapidly identify optimal stimulus parameters without *a priori* knowledge of the full frequency-amplitude activation map for new RGC populations. An interior point algorithm (Byrd et al., 1999; Waltz et al., 2006) was used to search for the optimal stimulus amplitude and frequency in 15 groups of virtual RGC populations. The resulting stimulation parameters are shown with mean ± *SD* in Table 2. These results suggest that preferential ON RGC activation can be best achieved at a stimulation amplitude of 155 ± 22 μA and frequency 6.25 ± 0.001 kHz. Similarly, preferential OFF RGC activation can be achieved at 65 ± 18 μA and 3.25 ± 2.59 kHz. These population-based simulation results are consistent with our *in vitro* observations—ON RGC types can be preferentially targeted at high stimulation amplitude and frequencies, whilst OFF RGCs can be preferentially targeted with lower stimulation amplitudes and a larger range of frequencies.

**Table 2:** Summary of the virtual and *in vitro* stimulation parameters for preferential ON and OFF RGC activation.

**Model performance (mean ±*SD*)**							
**Preferential ON activation**				**Preferential OFF activation**			
**Iterations**	**Measurements**	***F*** **(kHz)**	***A*** **(μA)**	**Iterations**	**Measurements**	***F*** **(kHz)**	***A*** **(μA)**
16 ± 3	61 ± 10	6.25 ± 0.001	155 ± 22	10 ± 4	44 ± 13	3.25 ± 2.59	65 ± 18

*“Iterations” is the number of searching iterations performed, “Measurements” represents the number of function evaluations in the simulation, and indicates the number of recordings in the in vitro experiments*.

In Table 2, the number of function evaluations (measurements) in the simulations on the virtual retinas is analogous to the number of individual ON and OFF RGCs recorded by whole-cell patch clamp *in vitro*. From 15 samples of simulated RGC populations, the optimal stimulation for preferential ON RGC activation can be found by testing 61 ± 10 pulse trains and 44 ± 10 pulse trains for preferential OFF RGC activation. For comparison, to derive these results *in vitro* would require testing 192 different pulse trains for each RGC.

**Figure 4:**
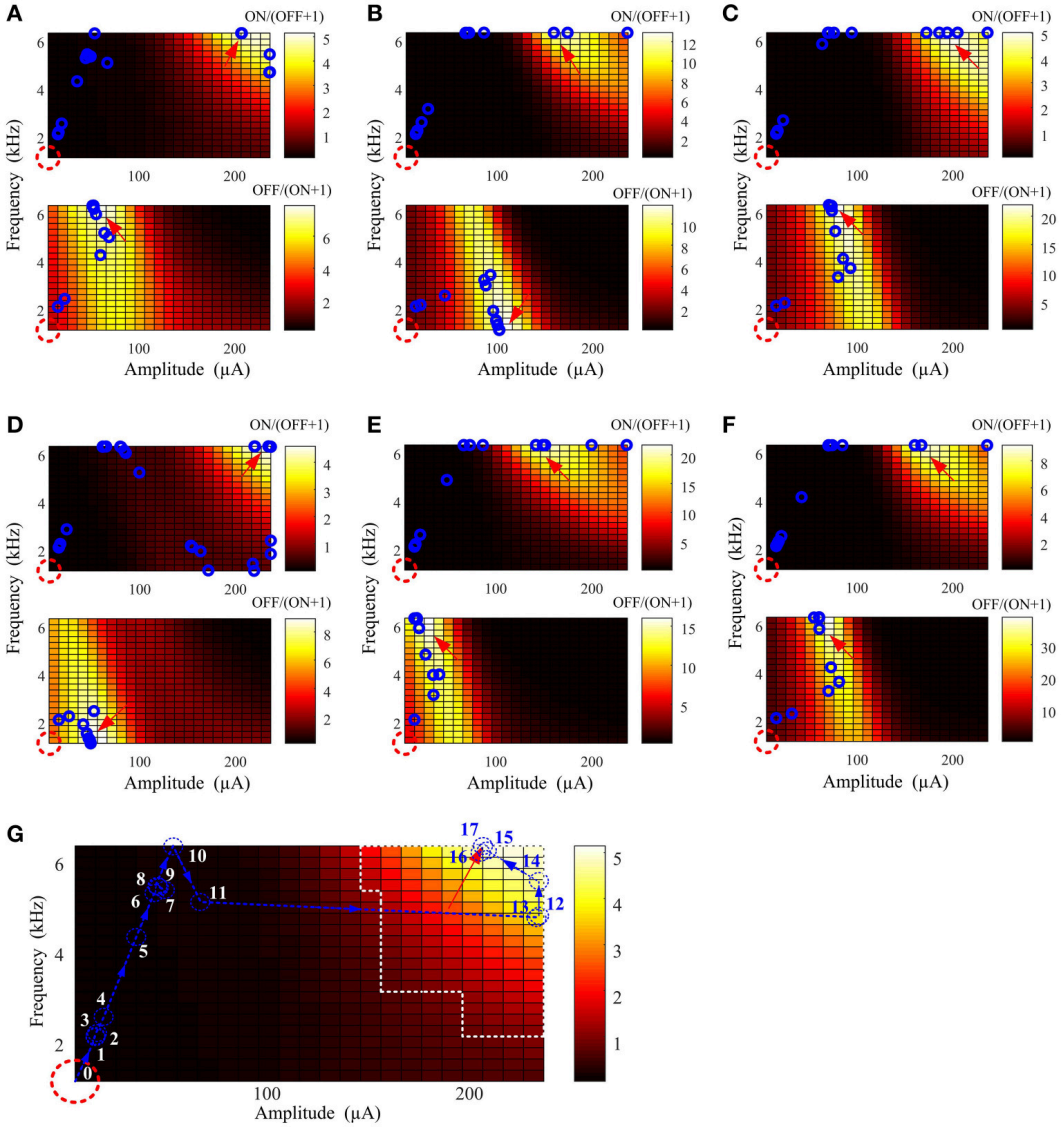
Optimal stimulation amplitude and frequency can be determined using a well-defined cost function, minimized with an interior point algorithm. **(A–F)** Six examples of parameter searching with different virtual ON and OFF populations. In each panel, the red dashed circle represents the starting point and the red arrow indicates the location where the search was terminated. Intermediate search points at each new iteration are represented by the blue circles. In all results, the optimal stimulus parameters were successfully found regardless of variations across the virtual RGC population. **(G)** An example search path. Each iteration is sequentially labeled from 0 (initial point) to 17 (final iteration). The region outlined by white dashed lines is the optimal stimulation parameter space in Figure 2C. The last six iterations (blue numbers) before the search was terminated were within the optimal parameter space defined by Table 2.

Figure 4 provides examples of stimulus parameter searching with six virtual ON and OFF populations. The starting point was indicated by a red dash circle and the optimal stimulus point by a red arrow. Each blue circle represents the stimulus parameters after a new searching iteration. Only 16 ± 3 and 10 ± 4 iterations were required to find the optimal stimulation parameters for ON and OFF activation, respectively. The optimal search path indicated by the few number of blue circles in each case indicates that stimulation parameters can be effectively found regardless of the physiological variation in different RGC populations. Finally, we found that in the last few iterations (blue circles around the optimal stimulus parameters for each case), the converged parameters were always within the optimal parameter range.

## Discussion

In this study, we have described an efficient algorithm for determining optimal stimulus parameters for preferential activation of RGC subtypes, for use in retinal neuroprosthetics. We began by evaluating the effects of high-rate stimulus amplitudes and frequencies on RGC responses *in vitro*. Our data suggested that: (1) preferential activation of either ON or OFF RGCs can be elicited by adjusting amplitudes and frequencies in HFS, and (2) HFS-based stimulation strategies may be helpful for mimicking the natural parallel RGC encoding. Finally, our *in silico* analysis on virtual RGC populations indicated that optimal HFS parameters could be rapidly identified in a closed-loop system using modeled neural responses in RGC subtypes as real-time feedback, without *a priori* knowledge of the full frequency-amplitude activation map for a new RGC population.

Improvements in the efficacy of neural prosthetic devices can stem from more sophisticated stimulation strategies which enable preferential activation of specific neuron types. Such a design would allow artificially evoked neural responses to more closely mimic specific aspects of physiological spiking patterns in response to natural inputs. Existing prosthetic neural devices, including retinal neuroprostheses, have limited ability to preferentially stimulate functionally-distinct RGCs. Studies such as ours, investigating the effects of stimulation parameters such as amplitude and frequency, are a necessary step to significantly improve the quality of elicited percepts, leading to improved performance of visual prostheses.

### Preferential activation of ON and OFF RGCs can be achieved over different ranges of HFS amplitudes and frequencies

ON and OFF pathways are the two core information streams in the primate retina, in which ON and OFF midget and parasol RGCs together significantly outnumber all remaining RGC types (Dacey et al., 2003). Discriminating between ON and OFF RGCs with electrical stimulation is therefore a vital first step toward improving artificial vision. Until recently, retinal stimulation has not been able to provide preferential activation of ON and OFF RGCs. Such co-activation is highly unnatural, providing conflicting information to higher visual centers, and potentially degrading the efficacy of retinal implants. Human subjects reported that evoked percepts, particularly for the low-electrode-count implants (Ayton et al., 2014), resembled halos, blobs, streaks, or other more complex patterns (Humayun et al., 2003; Rizzo et al., 2003; Yanai et al., 2007; Zrenner et al., 2011; Nanduri et al., 2012; Weitz et al., 2015; Sinclair et al., 2016). In most cases, the underlying cause(s) remains unknown. To address this problem, we require improved understanding of how different functional RGCs respond to artificial stimulation, and how optimal stimulus waveforms can be delivered using closed-loop feedback systems. Preferentially activating one RGC type over another means that HFS-induced signals can be interpreted and translated more accurately, corresponding to better natural responses.

In this study, we built on previous *in vitro* (Cai et al., 2011, 2013; Twyford et al., 2014) and *in silico* (Guo et al., 2014, 2015; Kameneva et al., 2016) studies, finding that preferential activation of RGC types can be achieved over a wide range of HFS parameters. In particular, ON RGC types can be targeted at relatively higher stimulation amplitudes (>150 μA) and frequencies (2~6.25 kHz), whilst OFF RGCs can be targeted with lower stimulation amplitudes (40~90 μA), across all tested frequencies (1~6.25 kHz). The stimulation strategy described here may be useful for mimicking natural encoding of RGC visual patterns. Specifically, ON ganglion cells showed an increase in spiking as stimulus current increased whilst OFF RGC responses were inhibited by the same stimulus, and vice versa (Figure 2E).

A recent clinical study suggested that stimulation amplitude contributes to both size and brightness of elicited phosphenes, while frequency contributes only to the brightness (Nanduri et al., 2012). However, only moderately low frequencies (≤12 Hz) and amplitudes (<10 μA) were tested. By examining a much wider range of HFS amplitudes and frequencies, we observed the opposing effects these stimulus parameters could elicit from the RGCs. When stimulated by the same frequency, ON and OFF RGCs both exhibited non-monotonic spike-stimulus profiles with differences in thresholds, resulting in different stimulus amplitude ranges for rising and falling phases. The precise mechanisms underlying this non-monotonic activation remains largely unknown. Stimulus-strength-dependent suppression in response to stimulation from single monophasic pulses, has been reported by Boinagrov et al. (2010). Boinagrov et al. (2012), who suggested sodium current reversal for being the reason of inhibition. In another study, Rattay (2014) proposed the anodal block theory, in which anodic surround of the focal cathodic pulse causing the nerve membrane on the outer wall of the pipette to become hyperpolarized by the current converging toward the tip of the electrode. However, further modeling and *in vitro* studies are still required to better understand the factors that shape the response of a retinal neuron to biphasic HFS. In particular, efforts should be devoted to assessing the contribution of intrinsic RGC properties in shaping RGC spiking profiles.

The HFS-induced differential RGC activations were also reported in prior studies (Cai et al., 2013; Twyford et al., 2014). In this study, we further found that increasing the frequency can enhance differential activation, maximizing the range of stimulation amplitudes that allowed for differential activation. In addition, both our *in vitro* and *in silico* results suggest that preferential activation of RGCs may be further improved by increasing the stimulation frequency over 6.25 kHz, given the larger stimulus current margin and lower threshold at high frequencies, as shown in Figure 2B. It should be noted that higher frequencies can sacrifice the stimulation efficacy (Cai et al., 2011; Hadjinicolaou et al., 2015). Therefore, a balance between a sufficient margin for preferential activation and a reasonable stimulation efficacy must be achieved.

### Closed-loop efficient searching for optimal stimulation parameters

In our *in vitro* experiments, a large amount of HFS candidate pulse trains are delivered to investigate the efficacy of each combination of stimulus parameters. In particular, 192 trials (8 levels of pulse frequency and 24 levels of pulse amplitude at each frequency) of biphasic pulse trains were delivered to each RGC. This process is time-consuming and potentially unattainable when a large stimulation parameter space (e.g., up to the frequency of 25 kHz) is to be evaluated, or if other pulse waveform shapes are to be considered (Hadjinicolaou et al., 2015; Raz-Prag et al., 2017). In addition, responses to HFS may be variable across functionally-distinct RGC types (Cai et al., 2011, 2013; Twyford et al., 2014) and a comprehensive description of electrically-evoked RGC responses is yet to be compiled, due to their large diversity in both intrinsic and morphological properties (O'Brien et al., 2002; Margolis and Detwiler, 2007; Wong et al., 2012). Therefore, we firstly developed a closed-loop searching algorithm to effectively find the optimal stimulation parameters without delivering all possible pulse trains. Secondly, we evaluated the performance of this searching algorithm against variable virtual RGC populations, to examine whether physiological variations could affect the utility of the algorithm.

Using modeled ON and OFF RGC spikes as real-time feedback, our closed-loop searching algorithm can (1) significantly shorten the exploration time for the optimal stimulus parameters without testing a large number of pulse trains *in situ*, and (2) robustly discover the optimal stimulus parameters regardless of the variations among RGC populations. Compared to non-optimized *in vitro* experiments (192 pulse trains), the numbers of spike measurements (61 ± 10 and 44 ± 10 pulse trains for exploring preferential ON and OFF RGC activation) are significantly reduced. Furthermore, the optimal search path indicated by the few number of searching iteration steps (16 ± 3 and 10 ± 4 iterations for exploring preferential ON and OFF RGC activation) in each case suggested that stimulation parameters can be robustly and effectively found regardless of the physiological variation in different RGC populations.

In addition, the performance of this searching algorithm can be affected by the form of objective function, which defines the best solution and the shape of the parameter searching surface. In this study, we found that the preferential activation can be best defined by simultaneously minimizing the electrically-evoked spike rate of one cell type whilst maximizing the spike rate of another type (i.e., $\frac{\sigma_{ON}}{\left(\sigma_{OFF}+1\right),\;}$ or $\frac{\sigma_{OFF}}{\left(\sigma_{ON}+1\right)\;}$). Our searching algorithm did not exhibit an equivalent level of performance with other objective functions, for example, the difference between the spike rate of cell types, did not exhibit an equivalent level of performance to the current (results not shown).

Since the major aim of this study is to investigate the optimal stimulus parameter space for preferential RGC ON/OFF activation, only eight frequency values were used to reconstruct the frequency-amplitude activation map for each RGC. This may lead to a “non-smooth” searching surface in practical parameter searching. Finer parameter search resolution is required in future experimental studies to reconstruct a smoother spike-stimulus surface function. In addition, practical optimal stimulation parameters are likely to involve a range of values, rather than a single optimal point (global minimum) as shown in Figure 3, due to additional considerations and trade-offs not explored here (e.g., range and resolution of stimulator frequency and amplitude, safety concerns, etc.). As demonstrated in each case in Figure 4, the last few (3~4) iterations before the searching terminated were always within the experimentally-identified optimal parameter range for preferential RGC activation, reinforce this assertion. In other words, finding a strict global minimum is not necessary for practical optimal parameter searching. These results suggested that our method can help experimentalists rapidly target the likely optimal space with minimal steps. As shown in Figure 2E, the optimal stimulus parameters can be quickly found, then verified with patch-clamp recordings.

It should be noticed that the stimulation parameters identified using our present technique may not be optimal for every particular neuron. However, by searching for the stimulation parameters in the population-averaged spike-stimulus surface, the identified stimulation parameters do elicit functionally useful level of preferential activation. Further *in vitro* experiments are still required to validate this method real-time through a combination of dual patch-clamp recordings (Tsai et al., 2017) and computer-supported optimization approaches.

## Future work

It remains to be seen if preferential RGC activation, as described here, can be used in practice. Here we will examine some issues that may arise when translating our findings to clinical settings. First, the size and the location of the stimulus electrodes used in existing HFS-based studies are far smaller and closer to the target neurons than those used in clinical devices. Second, the 200 μA stimuli used in this study is higher than the reported safe charge injection limits for electrodes fabricated from Pt-black or similar materials (Rose and Robblee, 1990). Stimulus charge density can be limited by relaxing other stimulus variables such as pulse-width duration or electrode size. Third, the current method only works with *in vitro* setups recording individual RGCs that have been identified by light stimuli. It could in future be incorporated into actual bionic eye devices, if a reliable method could be found to classify and record different RGC subtypes. This may be achieved by examining the spiking patterns of RGCs using implants with recording capabilities, as many OFF neurons exhibit spontaneous spikes (Margolis and Detwiler, 2007). At present, we only consider rate-codes in our objective functions. In the future, however, it may be possible to update these functions with known temporal features of the required spike coding patterns for preferential activation of one RGC type over another. Moreover, given that there are 30 different RGC subtypes, it is likely that the results of this study only represent specific ON and OFF subsets with large somas rather than the entire ON and OFF RGC population. Further *in vitro* and modeling studies are still required to validate the reliability and generalizability of preferential activation for a larger RGC population, which can potentially be achieved using high-density multielectrode arrays (Tsai et al., 2017) and calcium imaging (Weitz et al., 2015). Finally, electrical stimulation can activate not only RGCs, but potentially also any remaining neurons in the degenerated retinal network (Werginz and Rattay, 2016; Tsai et al., 2017). Our present results are limited to the contribution of synaptically isolated RGCs, without contributions from the retinal interneurons. In future, additional *in vitro* electrophysiology studies using degenerative models will allow us to better understand how RGC types could be preferentially targeted in the diseased retina.

## Author contributions

TG, CY, and DT: conceived of and designed the study; TG, CY, and MM: performed the *in vitro* experiments; TG and SD: performed the computational simulations; TG, CY, GS, JM, SD, and NL: analyzed the data. All authors drafted the manuscript, read and approved of the final manuscript.

### Conflict of interest statement

The authors declare that the research was conducted in the absence of any commercial or financial relationships that could be construed as a potential conflict of interest.
