# Supplementary material for: Closed-Loop Efficient Searching of Optimal Electrical Stimulation Parameters for Preferential Excitation of Retinal Ganglion Cells
Source: Front Neurosci. 2018 Mar 19;12:168. doi: 10.3389/fnins.2018.00168 (PMC5867314; doi:10.3389/fnins.2018.00168)
Supplement: Supplementary file 1 [file Image1.PDF]

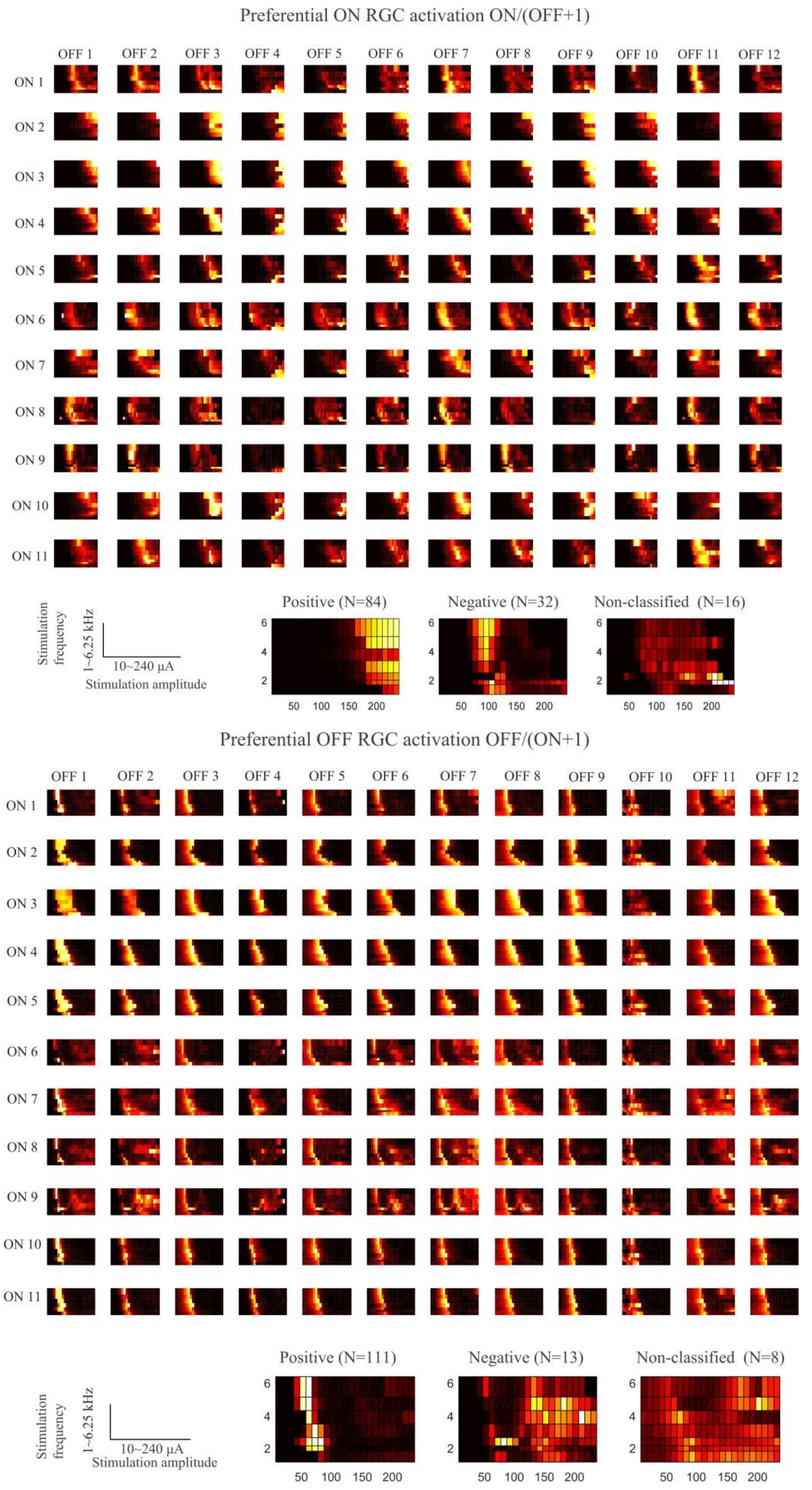

Supplementary Figure 1. Preferential activation maps for individual ON (N=11) and OFF RGC (N=12) pairwise. Preferential activation maps were classified as positive, negative, or non-classified. **Positive**: if the resulted optimal parameter space for preferential activation overlapped with the mean activation map of the same RGC population. **Negative**: if the resulted optimal parameter space overlapped with the mean activation

map calculated by the opposite RGC population. **Non-classified**: if the resulted optimal parameter space overlapped with the mean activation map of both or either population. An example of each cluster was given.

Additional results have been provided in the Supplementary Figure 1 below to show the normalized preferential activation map of individual ON and OFF RGC pairwise (11x12). Resulted preferential activation maps were further classified based on:

1) **Positive**: if the resulted optimal parameter space for preferential activation (defined as stimulation settings for which the overall spike number from one cell type is three times that of another cell type) overlapped with the mean activation map of the same RGC population.
2) **Negative**: if the resulted optimal parameter space overlapped with the mean activation map calculated by the opposite RGC population.
3) **Non-classified**: if the resulted optimal parameter space overlapped with the mean activation map of both or either population.

An example of each cluster was given in the bottom of the Supplementary Figure 1. In general, 84/132 of pairwise classified as positive for preferential ON activation maps, and 111/132 of pairwise classified as positive for preferential OFF activation maps. These results indicate that our searched optimal HFS parameters work for the vast majority of the RGCs.
